# Supplementary material for: Visual working memory models of delayed estimation do not generalize to whole-report tasks
Source: J Vis. 2024 Jul 26;24(7):16. doi: 10.1167/jov.24.7.16 (PMC11282892; doi:10.1167/jov.24.7.16)
Supplement: Supplement 1 [file jovi-24-7-16_s001.pdf]

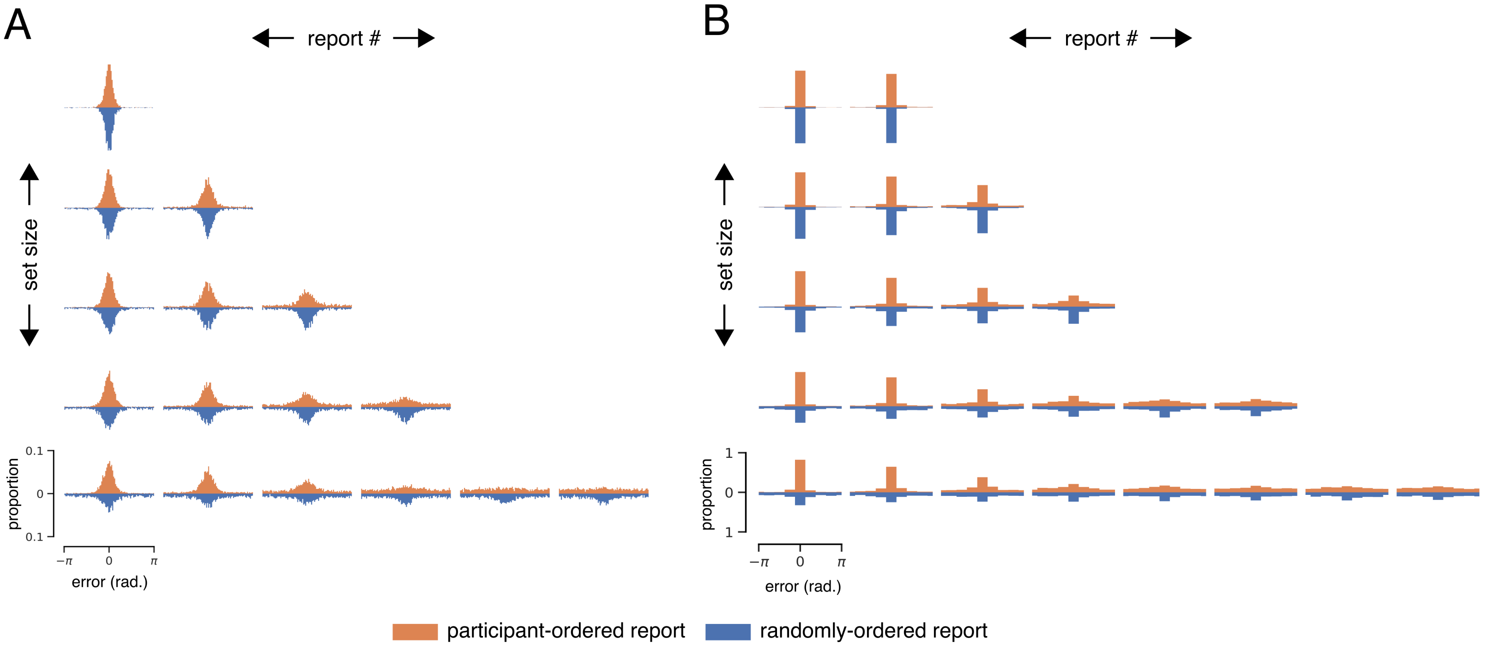

Supplementary Figure 1. Color report error distributions for all set sizes, separated by report order. Includes all participants. A Continuous error distributions. 360 possible error values divided into 90 bins for visualization. Orange and blue histograms show errors from participant-selected and randomly-generated report conditions, respectively. **B** Discrete error distributions. Same as A, but bins correspond to all possible error values.
