# Supplementary material for: Visual working memory models of delayed estimation do not generalize to whole-report tasks
Source: J Vis. 2024 Jul 26;24(7):16. doi: 10.1167/jov.24.7.16 (PMC11282892; doi:10.1167/jov.24.7.16)
Supplement: Supplement 2 [file jovi-24-7-16_s002.pdf]

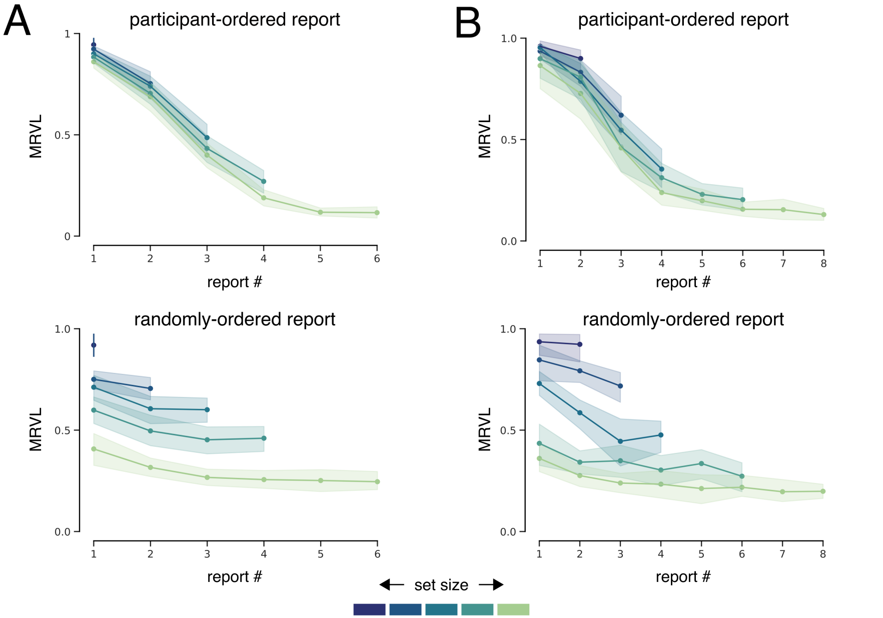

Supplementary Figure 2. Mean resultant vector length (MRVL) of color report error distributions, separated by report order. A Mean MRVL averaged across participants for the continuous whole report task. Shaded bars indicate +/- SEM. **B** Same as A, but for the discrete task.
