# Supplementary material for: Visual working memory models of delayed estimation do not generalize to whole-report tasks
Source: J Vis. 2024 Jul 26;24(7):16. doi: 10.1167/jov.24.7.16 (PMC11282892; doi:10.1167/jov.24.7.16)
Supplement: Supplement 3 [file jovi-24-7-16_s003.pdf]

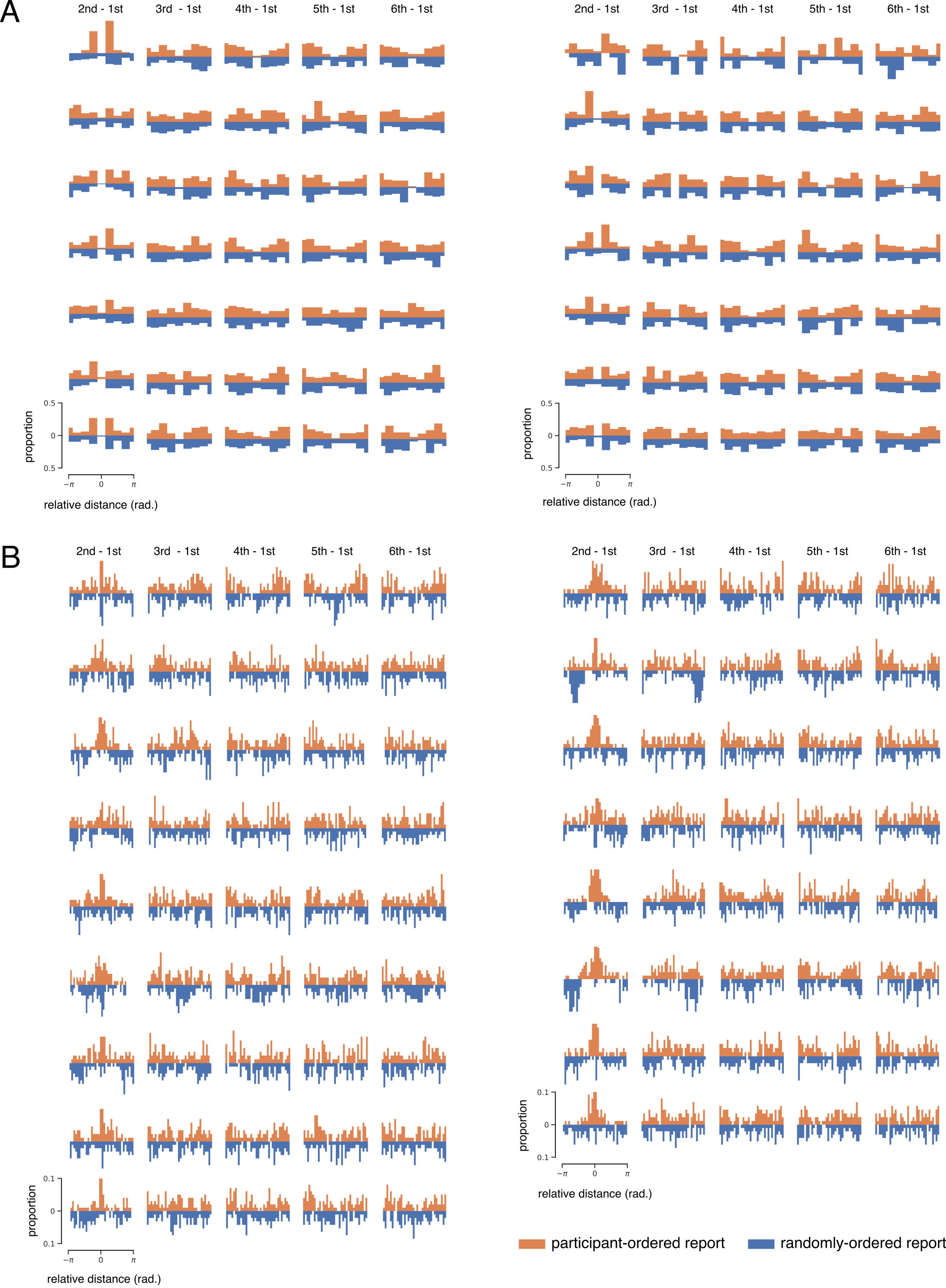

Supplementary Figure 3. Within-trial joint color report distributions separated by participant. A Distribution of relative distances for the discrete task (set size 6). Each row shows data for one participant. **B** Same as A, but for the continuous task.
