# Supplementary material for: Visual working memory models of delayed estimation do not generalize to whole-report tasks
Source: J Vis. 2024 Jul 26;24(7):16. doi: 10.1167/jov.24.7.16 (PMC11282892; doi:10.1167/jov.24.7.16)
Supplement: Supplement 4 [file jovi-24-7-16_s004.pdf]

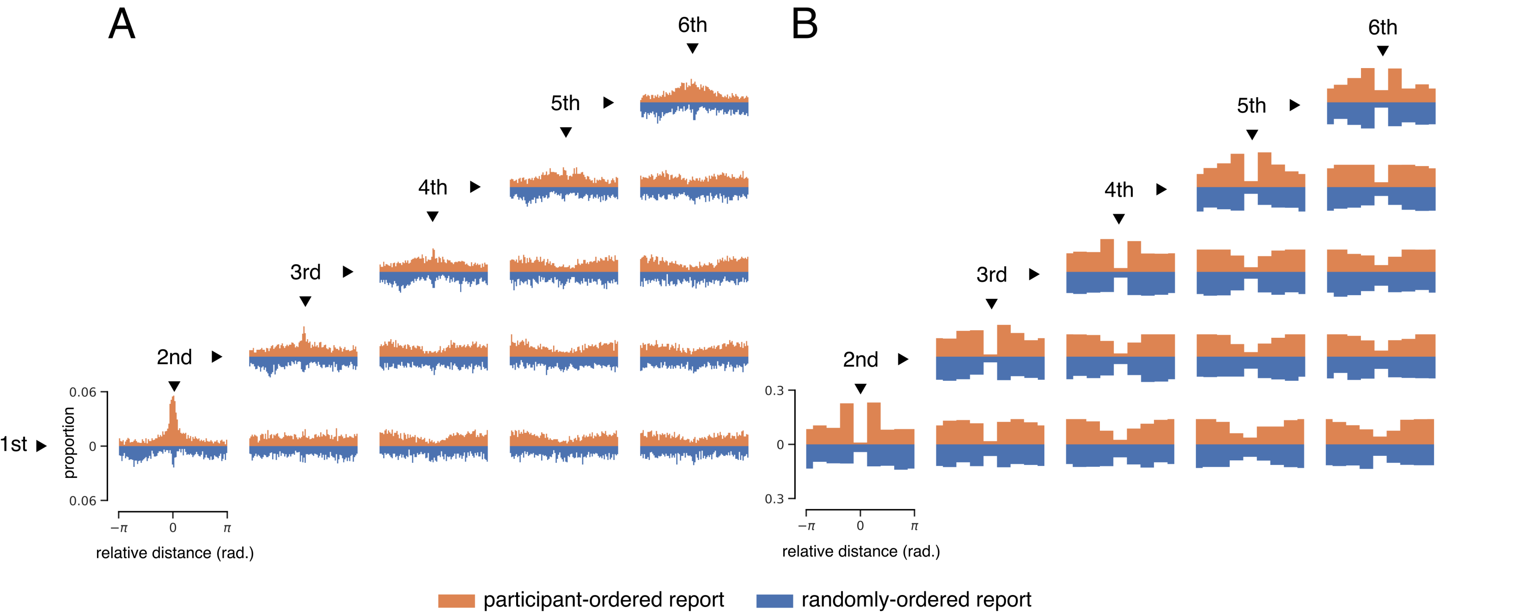

Supplementary Figure 4. Within-trial joint color report distributions for all set sizes, collapsed across participants. A Distribution of relative distances for the continuous task. Each row shows data for one set size. **B** Same as A but for the discrete task.
