# Supplementary material for: Visual working memory models of delayed estimation do not generalize to whole-report tasks
Source: J Vis. 2024 Jul 26;24(7):16. doi: 10.1167/jov.24.7.16 (PMC11282892; doi:10.1167/jov.24.7.16)
Supplement: Supplement 5 [file jovi-24-7-16_s005.pdf]

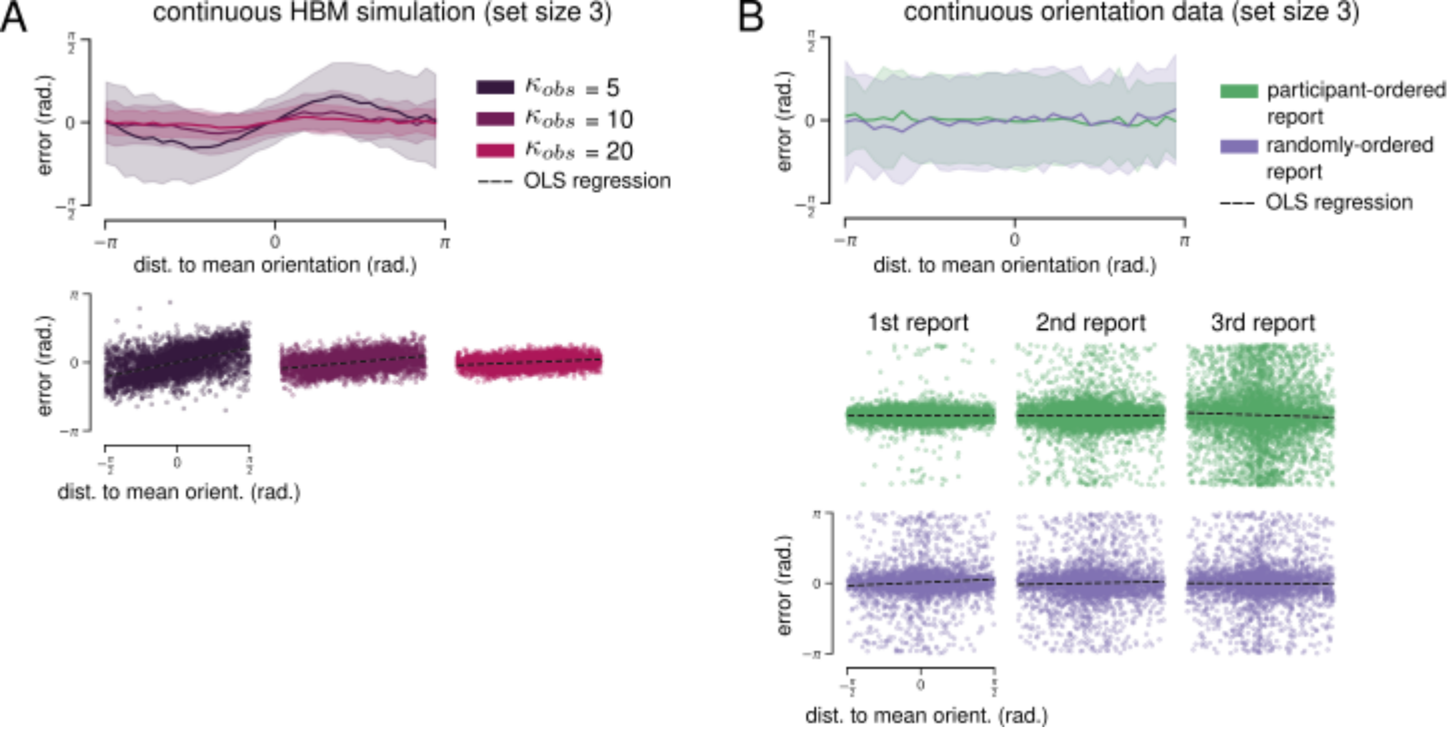

Supplementary Figure 5. Hierarchical Bayesian model (HBM) simulation results for continuous task with orientation stimuli. A *Upper*: Biases predicted by HBM simulations of the continuous task. Mean report error plotted as a function of the reported orientation's distance to the mean orientation. 360 error values are divided into 90 bins for visualization, and shaded area shows standard deviation. *Lower*: OLS regression of simulated report error on distance to mean orientation (dashed black lines). **B** *Upper*: Empirical biases for the continuous task at set size 3 (collapsed across all reports). *Lower*: OLS regression of empirical report error on distance to mean orientation. Each column shows results for a different report number.
