# Supplementary material for: Visual working memory models of delayed estimation do not generalize to whole-report tasks
Source: J Vis. 2024 Jul 26;24(7):16. doi: 10.1167/jov.24.7.16 (PMC11282892; doi:10.1167/jov.24.7.16)
Supplement: Supplement 6 [file jovi-24-7-16_s006.pdf]

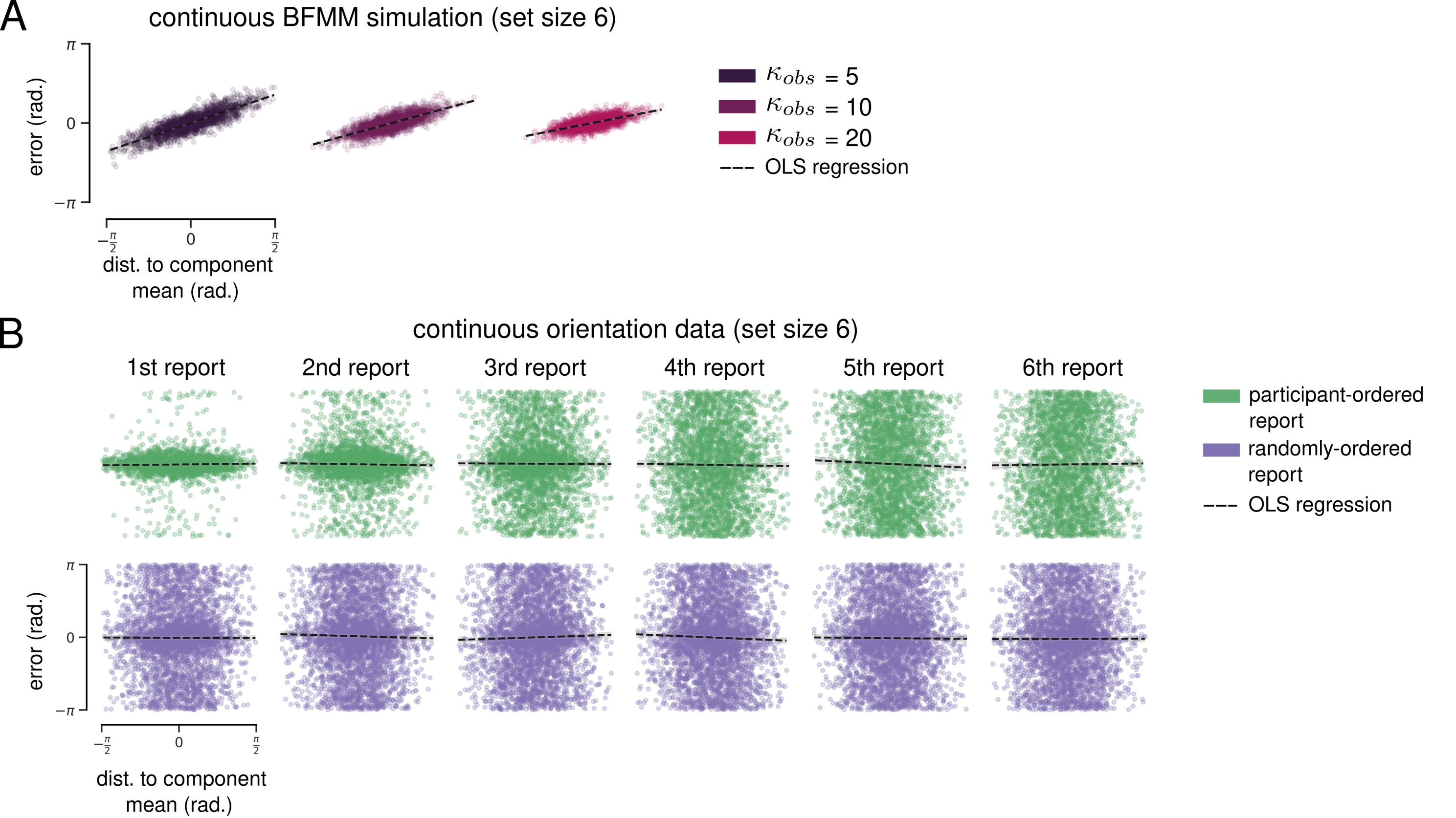

Supplementary Figure 6. Bayesian finite mixture model (BFMM) simulation results for continuous task with orientation stimuli. A *Upper*: Biases predicted by BFMM simulations of the continuous task. Mean report error plotted as a function of the reported orientation's distance to the component mean. 360 error values are divided into 90 bins for visualization, and shaded area shows standard deviation. *Lower*: OLS regression of simulated report error on distance to component mean (dashed black lines). **B** OLS regression of empirical report error on distance to component mean. Each column shows results for a different report number.
